# Effects of parenteral nutrition + best supportive nutritional care vs. best supportive nutritional care alone on quality of life in patients with pancreatic cancer—a secondary analysis of PANUSCO

**DOI:** 10.1007/s00520-024-08666-1

**Published:** 2024-06-27

**Authors:** Aline Emanuel, Friederike Rosenberger, Julia Krampitz, Christiane Decker-Baumann, Angela Märten, Dirk Jäger, Ingeborg Rötzer

**Affiliations:** 1 Division of Nutrition Sciences, German University of Applied Sciences for Prevention and Health Management (DHfPG), 66123 Saarbruecken, Germany; 2 Division of Health Sciences, German University of Applied Sciences for Prevention and Health Management (DHfPG), 66123 Saarbruecken, Germany; 3 Division of Psychology and Pedagogy, German University of Applied Sciences for Prevention and Health Management (DHfPG), 66123 Saarbruecken, Germany; 4 Department of Medical Oncology, National Center for Tumor Diseases (NCT), Heidelberg University Hospital, 69120 Heidelberg, Germany; 5 Boehringer Ingelheim International GmbH, Ingelheim Am Rhein, Germany; 6 Clinic for Oncology and Haemotology, Northwest Hospital, UCT-Cancer University Center, 60488 Frankfurt Am Main, Germany

**Keywords:** Parenteral nutrition, Quality of life, Pancreatic cancer, QLQ-C30, QLQ-PAN26

## Abstract

**Purpose:**

Parenteral nutrition (PN) can be an effective treatment to improve the nutritional status of patients with pancreatic cancer, but the effects of PN on quality of life (QoL) are still understudied. Therefore, we aimed at investigating whether the best supportive nutritional care (BSNC) in combination with PN at home compared to BSNC alone changed QoL in patients with advanced pancreatic cancer undergoing chemotherapy over a period of 7 weeks.

**Methods:**

*n* = 12 patients in the PANUSCO study received nutritional counseling only (control group (CG)) and *n* = 9 patients were also given supportive PN (intervention group (IG)). The primary endpoint was the change of QoL (EORTC-QLQ-C30 and QLQ-PAN26) over 7 weeks between the groups.

**Results:**

There was a significant worsening in social functioning in IG (*p* = 0.031) and a significant difference between groups in change of social functioning (*p* = 0.020). In all other domains of QoL, there was no significant difference between groups. Within groups, there was a significant improvement in the domain weight loss in IG (*p* = 0.031), showing that patients were less worried about their weight being too low. Furthermore, there was a significant difference in the change of BW over time between groups (*p* < 0.001) with IG showing an increase (*p* = 0.004) and CG showing no change (*p* = 0.578).

**Conclusion:**

The administration of PN had in one of five domains negative consequences on QoL. The decision to administer PN should always be made individually and together with the patient, and the impact on QoL should be included in the decision to administer PN.

**Supplementary Information:**

The online version contains supplementary material available at 10.1007/s00520-024-08666-1.

## Introduction

Pancreatic cancer (PaCa) showed an increasing mortality trend [1], often diagnosed at locally advanced or metastatic stage [2, 3], leading to weight loss (WL) [4, 5], tumor cachexia [6], malnutrition [7–10], and systemic inflammation [11]. WL leads to poor treatment outcome, shorter survival time [12–17], and reduced quality of life (QoL) [16]. Malnutrition is associated with increased chemotherapy (CTx)-induced toxicity, low treatment compliance, poor QoL, and overall survival [18–21].

Since the 1990s, there has been a growing interest in the QoL of cancer patients [22], acknowledging its impact on oncologic treatment compliance, prognosis, and mortality [23, 24]. Evidence supports the relationship between nutritional interventions (NI), nutritional status (NS), and QoL in cancer patients [25]. The importance of the NI in terms of QoL was also described in a systematic review, which showed that a NI might be associated with a better QoL in humans with different diseases [26]. Two further studies investigated QoL in cancer patients receiving parenteral nutrition (PN) at home and concluded that this form of PN was associated with an improvement in QoL [27, 28]. In contrast, an RCT showed that PN in advanced cancer had no impact on health-related QoL [29]. A further RCT in cancer patients showed that dietary supplements (+ usual diet) or a usual diet alone worsened QoL vs. an individualized nutritional counseling and education after 6.5 years [30].

Maintaining or improving NS is a key factor for QoL. Besides, nutritional counseling, enteral nutrition (EN), and PN are available interventions. EN is the administration of a special liquid food mixture through a tube into the gastrointestinal tract [31]. PN is the application of nutrients via a central venous access directly into the bloodstream, bypassing the digestive tract [31]. Previous studies showed an advantage of EN vs. PN in the treatment of PaCa (e.g., lower rate and development of complications and lower WL) [32] but EN frequently shows adverse events (e.g. nausea and vomiting) [33, 34]. Moreover, some patients do not wish to receive nasogastric tube feeding because of the psychological and social impact [35]. Therefore, PN is considered an alternative treatment for rapidly increasing energy intake (EI) with fewer adverse events on the gastrointestinal tract, but a higher risk for infectious complications [33, 34, 36]. The German Cancer Society recommends individualized nutritional assessment, nutritional counseling by dietitians, and the administration of supplemental or total EN or PN in cases of inadequate nutritional intake with the aim of maintaining or improving NS and QoL [37].

However, it remains understudied whether PN, while effectively increasing nutritional intake, has a negative impact on QoL in PaCa patients. A prospective observational study in participants with mixed conditions on the impact of PN on QoL showed a trend towards an improvement in the physical health component after 3 months, while the psychological component remained stable [38]. A systematic review of non-cancer patients criticized the lack of sufficient literature on the impact of PN on QoL [39]. To close this knowledge gap, this analysis aimed to investigate whether PN in combination with best supportive nutritional care (BSNC), compared to BSNC alone changed QoL in patients with advanced PaCa undergoing CTx over 7 weeks.

## Materials and methods

### Design

The data for this analysis are from the PANUSCO trial (NCT01362582). PANUSCO was a controlled, open-label, prospective, randomized, phase IIIb, multicenter trial with two parallel arms to investigate the effects of PN (intervention group (IG)) in combination with BSNC versus BSNC alone, (control group (CG)) on event-free survival in patients with advanced PaCa [40] (Fig S I).

The study was terminated prematurely because the calculated sample size of 120 patients could not be achieved. In this secondary analysis, the available datasets from four centers (Heidelberg, Erlangen, Bad Soden, Greifswald—Germany) were evaluated in an exploratory way. Data sets from weekly visits from baseline (*t*0) to week 7 (*t*7) were analyzed (our baseline represents *t*1 of the study protocol). The primary endpoint of this exploratory analysis was the change of QoL over 7 weeks between groups. Furthermore, correlations between changes in EI as well as protein intake (PI) and changes of QoL were investigated for all participants irrespective of group allocation.

### Participants

Patients over 18 years of age with histologically confirmed advanced pancreatic adenocarcinoma who had received at least one prior CTx (gemcitabine-based) and experienced disease progression on this prior CTx were screened for participation. At enrolment, all patients received 5-fluorouracil (5-FU), folinic acid (FA), and oxaliplatin as second or higher line CTx [40]. QoL-related inclusion criteria were a body mass index (BMI) ≥ 19 kg/m^2^, an expected life expectancy of more than 3 months, and at least one previous CTx. QoL-related exclusion criteria were a major surgery of less than 4 weeks prior to enrolment, a WL of more than two percentage within the last 7 days or an EI less than 500 kcal expected within the next 5 days, and more than 4 weeks of PN within the last 6 months or PN less than 4 weeks prior to enrolment. Detailed inclusion and exclusion criteria are given in [40]. Patients were randomly assigned to IG or CG.

### Nutrition of the patients

Both, the IG and CG received BSNC, defined as weekly nutritional consultation (face-to-face or by phone) and recommendation by experienced nutritionists. All types of oral nutritional supplements were allowed. Independent of oral food intake, the IG additionally received a defined, supportive PN. Defined stop criteria for nutritional therapy, e.g., energy intake of at least 50% of requirements, ensured that an adequate supply of nutrients was guaranteed for both the CG and IG. The infusions contained 1150 kcal, 50 g amino acids, 125 g glucose, and 38 g (+ 5 g fish oil extra) fat with soybean oil, medium chain triglycerides, olive oil, and fish oil. PN was planned to be given continuously overnight, 6 days per week at home. The actual administration of PN was recorded. The parentally prescribed EI was the same for all participants and did not depend on body weight (BW) or NS. This was not a total PN, but supplemental PN for all. Detailed information of PN is described in [40].

The NI as well as the CTx was administered until individual discontinuation criteria were met. For both arms, there were individual stopping criteria for the NI (Tab S I).

### Quality of life

The European Organization for Research and Treatment of Cancer (EORTC) quality of life questionnaire (QLQ-C30) and its supplementary module for PaCa QLQ-PAN26 in the validated German translation were used for assessing health-related QoL. A scoping review showed that the QLQ-C30 questionnaire was the most commonly used disease-specific instrument for QoL assessment in patients with PN and EN [41]. The QLQ-C30 with 30 questions is composed of both multi-item scales and single-item measures [42]. The questionnaire consists of five functional scales (physical, role, emotional, cognitive, and social functioning), three symptom scales (fatigue, nausea/vomiting, and pain), a global health status/ QoL scale, and six single items (dyspnea, insomnia, appetite loss, constipation, diarrhea, and financial difficulties) [42]. The domain role functioning asks if there were any limitations during work or leisure. The QLQ-PAN 26 includes 26 questions. It consists of seven scales, five of which are symptom scales (pancreatic pain, digestive symptoms, altered bowel habit, hepatic symptoms, and body image) and two functional scales (satisfaction with health care and sexuality). In addition, 10 single items are surveyed (symptom items: bloating, taste, indigestion, flatulence, WL, weakness arms and legs, dry mouth, trouble with side effects, future worries, and planning of activities). The scale of digestive symptoms asks whether there were any limitations in food selection and quantity due to the disease or treatment. The item WL asks if patients are concerned about low BW. There are four answer options (not at all, a little, quite a bit, very much) for each question in both questionnaires. All scales and single items are transformed to a scale from 0 to 100 [42]. A high score in the functional scale represents a high/healthy level of functioning [42]. A high score in global health status/QoL represents a high QoL [42]. However, a high score in symptom scale/item represents a high level of symptomatology/problems [42]. The different domains of the QLQ-C30 and QLQ-PAN26 were evaluated separately.

### Sample size

A total of 31 patients were enrolled in PANUSCO at four centers, and data on QoL were available for 30 patients. Data from patients who participated in the study for at least 7 weeks were analyzed. Therefore, 21 patients (IG: *n* = 9; CG: *n* = 12) were included in this secondary analysis.

After the patient’s eligibility for randomization was assessed, patients were randomly assigned to one of the two treatment arms (1:1) Stratification was performed prior to randomization by Eastern Cooperative Oncology Group Performance Status (ECOG PS) (stratum 1: PS < 2, stratum 2: PS ≥ 2) [40].

### Statistical analysis

Descriptive statistics (means and standard deviations) were performed for patient characteristics and for all items of QLQ-C30 and QLQ-PAN26. For missing data of patient characteristics, the last available data were imputed. For single missing items of QLQ-C30 and PAN26, they were rated as missing, as recommended in the EORTC manual [42]. However, only one item of one patient in the QLQ C30 questionnaire was missing. In QLQ-PAN26, two items were missing in four patients. These included questions on sexuality, which, according to the manual, can often lead to problems with the questionnaire [42]. The data set did not meet the requirement for parametric statistical procedures. Therefore, Wilcoxon tests were performed for all items of QLQ-C30 and QLQ-PAN26 for each group to test for changes over the course of the 7-week intervention. Mann–Whitney *U*-Tests were performed for all items of QLQ-C30 and QLQ-PAN26 to test for differences between the groups at baseline (*t*0), at the end of the analysis period (*t*7), and for change from *t*0 to *t*7 (delta). Spearman’s rank correlation test was performed to test the correlation between the changes of all items of (a) QLQ-C30 and (b) QLQ-PAN26 with the changes in PI in g/kg BW, the changes in EI, and the changes in BW from *t*0 to *t*7 for all participants irrespective of group allocation. All tests were performed with exact significance and were two-tailed. *p* < 0.05 indicates statistical significance. The IBM SPSS version 28.0 software, Chicago, IL, USA, was used.

## Results

### Patients’ characteristics and nutrition

The clinical characteristics of the patients are shown in Table 1. There were no significant baseline differences between groups except for BW, BMI, and EI. Over the course of the 7-week intervention, IG had a significantly higher mean EI and PI in g/kg BW incl. PN. Furthermore, there was a significant difference in the change of BW over time between groups (*p* < 0.001) with IG showing an increase (*p* = 0.004) and CG showing no change (*p* = 0.578). There were no catheter-related infections in either group.

**Table 1:** Patients’ characteristics (*t*0)

	**Intervention, *n* = 9**	**Control, *n* = 12**	***p***
Gender (M/F)	5/4	6/6	1.000^1^
Age (years)	65 ± 6.6	68 ± 6.0	0.283^2^
Body weight (kg)	62.3 ± 10.6	67.2 ± 13.5	0.464^2^
Ø Body weight *t*0-*t*7 (kg)	63.88 ± 11.09	66.96 ± 13.56	0.687^2^
Change body weight *t*0 to *t*7 (kg)	3.16 ± 1.97	− 0.57 ± 2.28	** < 0.001**^**2**^
Body mass index (kg/m^2^)	21.4 ± 2.0	24.1 ± 2.8	**0.036**^**2**^
Phase angle (°)	4.91 ± 0.73	4.57 ± 0.83	0.431^2^
ECM/BCM ratio	1.24 ± 0.18	1.36 ± 0.32	0.686^2^
Handgrip strength (kg)	31.72 ± 8.31	26.08 ± 9.37	0.164^2^
Biceps size (cm)	24.93 ± 3.0	26.79 ± 3.00	0.105^2^
CRP (mg/L)	14.13 ± 10.10	10.83 ± 12.98	0.369^2^
mGPS 0 (*n*)	4	7	0.835^1^
mGPS 1 (*n*)	4	3	
mGPS 2 (*n*)	1	2	
Surgery (yes/no)	7/2	9/3	1.000^1^
Energy intake (kcal/d)	2715 ± 557	2006 ± 697	**0.028**^**2**^
of which PN (kcal)	935 ± 176	-	**-**
Ø Energy intake *t*0-t*7* incl. PN (kcal/d)	2607 ± 469	2078 ± 601	**0.041**^2^
Protein intake (g/d)	110.1 ± 31.2	80.5 ± 34.3	0.082^2^
of which PN (g)	40.7 ± 7.6		**-**
Ø Protein intake *t*0-*t*7 incl. PN (g/d)	108.7 ± 22.8	86.4 ± 31.2	0.129^2^
Ø Protein intake *t*0-*t*7 incl. PN (g/kg BW/d)	1.7 ± 0.3	1.3 ± 0.4	**0.026**^**2**^

Values are given as mean ± S.D., except for mGPS, gender (males (M) and females (F)), and surgery. ^1^Pearson-Chi-Square; ^2^Mann-Whitney *U*-Test

Data in bold emphasis indicate significant results

## Quality of life

### QLQ-C30

**Fig. 1:**
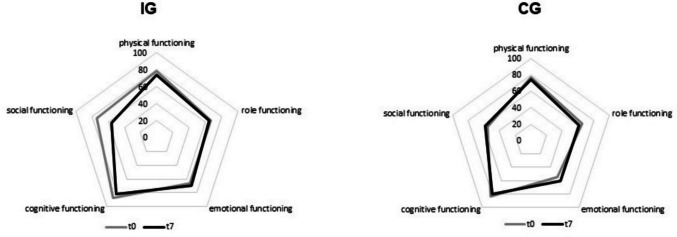
QLQ C30 functional scales at *t*0 (grey line) and *t*7 (black line) in IG and CG

**Fig. 2:**
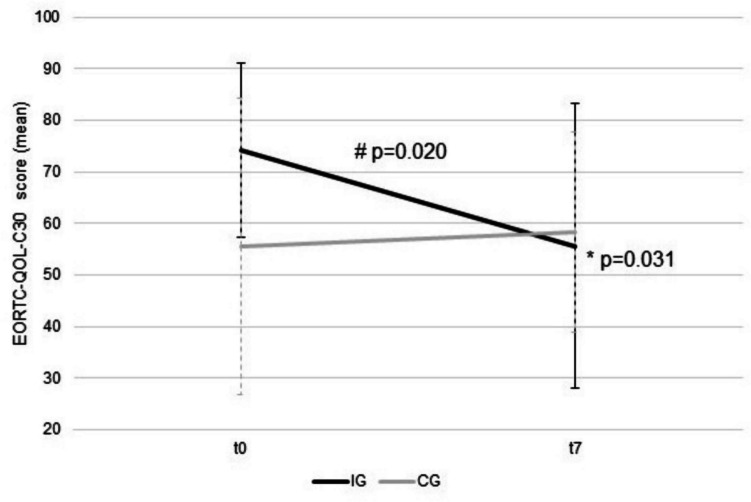
QLQ C30 change of social functioning (#differences between the groups for change from *t*0 to *t*7) and (* within IG) from *t*0 and *t*7 in IG (black) and CG (grey) (mean ± S.D.)

All QLQ-C30 functional scales of both groups are shown in Fig. 1. Change of social functioning scale in IG and CG is shown in Fig. 2.

There was a significant worsening in social functioning in IG from *t*0 to *t*7 (*p* = 0.031, Fig. 2, Table 2). Furthermore, the change in social functioning was different between the groups (*p* = 0.020, Fig. 2, Table 2). Financial difficulties were different between groups at *t*0 (*p* = 0.045). In other scales, there were no differences between groups and no changes from *t*0 to *t*7 within groups (Table 2).

**Table 2:** Changes in quality of life (QLQ-C30 questionnaire)

	**IG**		***p***^**1**^	**CG**		***p***^**1**^	***p***^**2**^	***p***^**3**^	***p***^**4**^
	*t*0	*t*7		*t*0	*t*7				
Global health status	62.04 ± 19.59	61.11 ± 21.65		59.03 ± 19.61	55.56 ± 21.71		0.792	0.510	0.491
**Functionality**									
Physical functioning	78.52 ± 20.21	74.07 ± 21.20	1.000	77.50 ± 19.85	74.44 ± 16.29	0.578	0.845	0.901	0.923
Role functioning	66.67 ± 34.36	64.81 ± 22.74	0.906	65.28 ± 32.92	61.11 ± 28.72	0.586	0.903	0.718	1.000
Emotional functioning	65.74 ± 28.40	69.44 ± 21.25	0.813	54.86 ± 22.88	61.11 ± 18.23	0.352	0.334	0.209	0.873
Cognitive functioning	87.04 ± 13.89	81.48 ± 17.57	0.250	83.33 ± 18.80	80.56 ± 17.16	0.750	0.745	0.935	0.587
Social functioning	74.08 ± 16.90	55.56 ± 27.64	**0.031**	55.56 ± 28.72	58.33 ± 19.46	0.844	0.136	0.971	**0.020**
**Symptoms**									
Fatigue	50.62 ± 30.99	39.51 ± 21.60	0.328	36.11 ± 22.29	41.67 ± 19.61	0.250	0.276	0.788	0.082
Nausea and vomiting	12.96 ± 13.89	20.37 ± 21.69	0.563	6.95 ± 15.01	12.50 ± 18.97	0.312	0.223	0.307	0.666
Pain	37.04 ± 36.11	31.48 ± 28.19	0.250	27.78 ± 31.25	22.22 ± 35.77	0.313	0.602	0.335	0.916
Dyspnea	25.93 ± 32.40	14.81 ± 24.22	0.500	27.78 ± 27.83	24.24 ± 26.21	1.000	0.878	0.501	0.251
Insomnia	37.04 ± 38.89	22.22 ± 33.33	0.250	36.11 ± 36.12	33.33 ± 31.78	0.656	0.991	0.366	0.262
Loss of appetite	29.63 ± 35.14	44.44 ± 28.87	0.742	25.00 ± 25.13	27.78 ± 34.33	1.000	0.890	0.211	0.638
Constipation	0 ± 0	0 ± 0	1.000	11.11 ± 29.59	5.56 ± 12.97	0.750	0.486	0.486	0.892
Diarrhea	44.45 ± 37.27	22.22 ± 23.57	0.094	22.22 ± 29.59	22.22 ± 32.82	1.000	0.198	0.821	0.137
Financial difficulties	0 ± 0	7.41 ± 22.22	1.000	22.22 ± 32.82	27.78 ± 31.25	0.500	**0.045**	0.125	0.683

Values are given as mean ± S.D. ^1^Wilcoxon Test for changes from *t*0 to *t*7 within group; ^2^Mann-Whitney *U*-Test for differences between the groups at *t*0; ^3^Mann-Whitney *U*-Test for differences between the groups at *t*7; ^4^Mann-Whitney *U*-Test for changes of QoL between the groups from *t*0 to *t*7

Data in bold emphasis indicate significant results

**Fig. 3:**
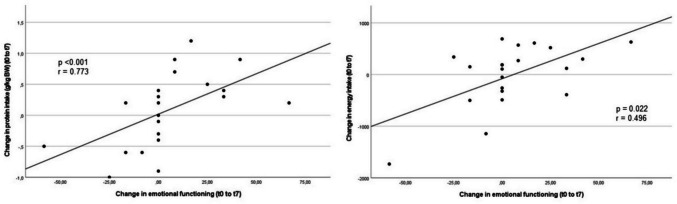
QLQ C30 correlations between change in emotional functioning and (1) change in protein intake as well as (2) change in energy intake

Correlations for all participants without consideration of group allocation are shown in Fig SII and SIII. An increase in PI (*p* < 0.001; *r* = 0.773; Fig. 3 (1)) or EI (*p* = 0.022; *r* = 0.496; Fig. 3 (2)) was associated with an improvement in emotional functioning. Furthermore, an increase in PI (which in contrast to the group-based analysis above was irrespective of the way of administration) was associated with an improvement of social functioning (*p* = 0.008; *r* = 0.562; Fig SII). Furthermore, there was a trend towards a correlation between an increase in EI and an improvement in role functioning (*p* = 0.066; *r* = 0.409; Fig SIII). In addition, there were no significant correlations between change in BW and change in the QLQC30 scales.

### QLQ-PAN26

Findings of the QLQ-PAN26 are presented in Table 3. There was a significant improvement in the domain WL in IG from *t*0 to *t*7 (*p* = 0.031; Table 3), showing that patients were less worried about their weight being too low. Satisfaction with health care was significantly higher in IG versus CG at *t*0 (*p* = 0.045; Table 3). Other symptom and functional scales showed no differences between groups and no changes from *t*0 to *t*7 within groups (Table 3). Delta values from *t*0 to *t*7 do not differ between groups (Table 3).

**Table 3:** Changes of quality of life (QLQ-PAN26 questionnaire)

	**IG**		***p***^1^	**CG**		*p*^1^	*p*^2^	*p*^3^	*p*^4^
	*t*0	i7		*t*0	*t*7				
**Symptom scales/items**									
Pancreatic pain	36.11 ± 34.61	30.56 ± 27.32	0.438	27.78 ± 27.83	25.23 ± 25.39	0.648	0.585	0.763	0.680
Bloating	40.74 ± 32.40	51.85 ± 37.68	0.375	30.55 ± 30.01	38.89 ± 34.33	0.219	0.519	0.498	1.000
Digestive symptoms	35.19 ± 37.68	46.30 ± 33.10	0.672	31.94 ± 31.35	26.39 ± 24.06	0.477	0.763	0.153	0.492
Taste	29.63 ± 42.31	14.81 ± 17.57	0.375	25.00 ± 28.87	16.67 ± 17.41	0.500	0.850	1.000	0.763
Indigestion	33.33 ± 40.83	22.22 ± 28.87	0.375	22.22 ± 29.59	33.33 ± 31.78	0.500	0.634	0.425	0.138
Flatulence	48.15 ± 41.20	37.04 ± 30.93	0.500	41.67 ± 32.18	50.00 ± 30.15	0.688	0.770	0.477	0.250
Weight loss	62.96 ± 38.89	37.04 ± 35.14	**0.031**	33.33 ± 37.60	41.67 ± 37.94	0.602	0.097	0.825	0.076
Weakness arms and legs	37.04 ± 26.06	25.92 ± 22.22	0.500	25.00 ± 28.87	33.33 ± 28.43	0.250	0.367	0.579	0.130
Dry mouth	48.15 ± 44.44	25.93 ± 32.40	0.422	27.78 ± 31.25	33.33 ± 34.82	0.594	0.390	0.656	0.335
Altered bowel habit	50.00 ± 38.19	35.18 ± 30.56	0.188	40.28 ± 27.94	43.06 ± 28.83	0.637	0.514	0.568	0.090
Hepatic	14.82 ± 25.61	11.11 ± 18.63	0.813	9.72 ± 19.41	5.56 ± 14.79	0.750	0.698	0.529	0.895
Body image	33.33 ± 20.41	38.89 ± 22.05	0.750	36.11 ± 28.28	37.50 ± 28.54	0.859	0.995	0.765	0.483
Troubled with side effects	33.33 ± 16.67	37.04 ± 30.93	0.750	45.45 ± 30.82	47.22 ± 26.43	1.000	0.302	0.368	1.000
Future worries	70.37 ± 30.93	59.26 ± 36.43	0.312	83.33 ± 26.59	72.22 ± 23.93	0.125	0.333	0.466	0.765
Planning of activities	55.56 ± 28.87	44.44 ± 23.57	0.500	47.22 ± 33.21	47.22 ± 36.12	0.813	0.522	0.938	0.643
**Functional scales**									
Satisfaction with health care	100.00 ± 0.00	100.00 ± 0.00	1.000	84.72 ± 28.83	91.67 ± 19.46	0.625	**0.045**	0.229	0.493
Sexuality	55.56 ± 49.30	74.07 ± 37.37	0.250	29.63 ± 42.31	40.74 ± 33.45	0.438	0.349	0.070	1.000

Values are given as mean ± S.D. ^1^Wilcoxon Test for changes from *t*0 to *t*7 within group; ^2^Mann-Whitney *U*-Test for differences between the groups at *t*0; ^3^Mann-Whitney *U*-Test for differences between the groups at *t*7; ^4^Mann-Whitney *U*-Test for changes of QoL between the groups from *t*0 to *t*7

Data in bold emphasis indicate significant results

Correlations for all participants without consideration of group allocation are shown in Fig SIV and SV. With increasing PI (*p* = 0.035, *r* =  − 0.461; Fig S IV (1)) and increasing EI (*p* = 0.018, *r* =  − 0.512; Fig SIV (1)), there was a worsening in planning of activities. Moreover, with increasing PI (*p* = 0.082, *r* = 0.388) and increasing EI (*p* = 0.097, *r* = 0.372), there was a trend towards worsening in indigestion, which means that these people had fewer digestive disorders. Furthermore, an increase in PI was correlated with an increase in side effects of treatment (*p* = 0.018, *r* = 0.523; Fig SIV (4)), but a trend towards a decrease in pancreatic pain (*p* = 0.055, *r* =  − 0.425). Moreover, there was a worsening in flatulence with an increase in EI (*p* = 0.024, *r* = 0.491; Fig SIV (2)). In contrast, digestive symptoms significantly decreased with an increase in PI (*p* = 0.040, *r* =  − 0.451; Fig SIV (2)) and there was a trend towards a reduction with an increase in EI (*p* = 0.090, *r* =  − 0.379), which means that there was a significant decrease in limitations in food choice and food quantity due to disease or treatment. The satisfaction with health care improved significantly with an increase in PI (*p* = 0.001, *r* = 0.664; Fig SIV (3)) and there was a trend for an improvement with an increase in EI (*p* = 0.076, *r* = 0.396). In addition, there were no significant correlations between change in BW and change in the PAN26 scales except for altered bowel habit which decreased with weight gain (*p* = 0.036, *r* =  − 0,459).

## Discussion

The aim of this secondary analysis was to investigate whether PN in combination with BSNC compared to BSNC alone changed the QoL in advanced PaCa patients undergoing CTx over 7 weeks. There was a significant worsening in social functioning in IG after the 7 weeks with a significant difference between the groups in change of social functioning from baseline to *t*7. No other between-group differences were found. In within-groups, there was a significant improvement in domain WL in IG, showing that patients were less worried about their weight being too low.

Correlations between PI and EI (independent of the group) showed that an increase in PI and EI was associated with an improvement in emotional functioning, digestive symptoms (PI only) and satisfaction with health care (EI only). Conversely, an increase in PI and EI correlated with a worsening in planning of activities, an increase in side effects of treatment (PI only), and a worsening of flatulence (EI only).

Initially, both groups showed a good NS with a significant difference in BMI between the groups. However, other parameters of NS, such as BW, phase angle, ECM/BCM ratio, handgrip strength, and bicep size, did not differ between the groups, so it can be assumed that our results were not influenced by the individual value of BMI. Baseline EI was also significantly higher in IG vs. CG, affected by PN which began at the same time. Unfortunately, data of EI and PI were unavailable for the time before the start of PN. Throughout the intervention, the mean EI and PI (incl. PN) and the change of BW were significantly higher in IG vs. CG as expected when administering PN.

Analyzing QLQ-C30 questionnaire results, social functioning decreased significantly in IG, consistent with an older trial in 19 patients with mixed conditions showing that patients receiving total PN lost their social status [43]. Eating is a part of the routine of daily life. A coffee break, a visit to a restaurant, or a meal with friends are part of everyone’s social life [43]. Patients who spend a lot of time at home because of PN can feel isolated and even lose friends [43]. Other factors of social life that were not asked about in the EORTC QLQ-C30 may also have been influenced. PN, involving invasive procedures, may contribute to social isolation. Although concerns about BW decreased, patients on PN can experience limitations in daily activities, affecting social life and autonomy. The aim of nutrition therapy is to ensure the supply of nutrients and fluids to improve QoL and medical outcomes. Typically, PN at home is administered by a nursing service, overnight, with infusion times of more than 10 h. Dependence on delivery and care processes limits patients’ autonomy, with consequences for their social life. Informing patients about PN aims and limitations of social functioning before treatment is crucial. However, other QLQ-C30 scales showed no changes over time. This is not in line with a prospective observational trial of 70 patients with cancer and other conditions who received PN and were interviewed for 2 years every 6 months with the SF-36v2 questionnaire. The study showed that there was an improvement in physical health component summary scores (comparable with domain physical functioning QLQ-C30) when patients got PN [38]. Reasons for this difference could be that this study differs in intervention length, patient group (cancer and non-cancer patients, no palliative therapy), and the survey instrument. A further reason could be that PaCa patients have a poor prognosis and rarely improve physical health over time. Notably, there was no difference in domain appetite loss between the groups, a positive aspect for the IG, because PN is often criticized for potential loss of appetite. Overall, changes in all but one QLQ-C30 scale in our trial did not show significant differences between groups, which can be interpreted as only little harm of PN on QoL in PaCa patients.

Analyzing QLQ-PAN26 questionnaire results, there were no group differences, but a decrease in worrying about their own WL in IG, as a result of PN. This is in line with the change of BW within intervention time, which increased significantly in the IG (Table 1). On the other hand, PN leads to a deterioration in social life. In addition to nutritional aims, both social stress and relief in the area of eating should be considered when deciding to PN. Satisfaction with health care was significantly higher in IG vs. CG at baseline when PN had recently started, likely due to increased contact with nurses during home-based PN. Other scales showed no differences between groups and no changes from baseline to *t*7 within groups. The decision-making process should be made together with the patient, acknowledging both positive and negative consequences of PN.

We examined PI and EI correlations with QoL in general, independent of group allocation or food administration. In QLQ-C30 increased PI and EI correlated significantly with an improvement in emotional functioning. Unlike group-based findings, increased PI showed an improvement of social functioning, suggesting that intake is crucial, but the method influences the results.

Regarding the QLQ-PAN26 questionnaire, we found significant correlations between an increase in PI and EI and a worsening in planning of daily activities. Furthermore, there was a significant correlation between an increase in PI and an increase in side effects of treatment. Our first analysis of PANUSCO showed no benefit of a high PI (> 1.5 g/kg/BW) irrespective of group allocation on NS and clinical outcomes. The worsening of the planning of daily activities can be explained by the fact that there were more patients with PN in the group with a high PI and thus supports the results from the QLQ-C30. Since the question about the occurrence of side effects is unspecific, a clear allocation is not possible. A previous data analysis showed a significantly lower incidence of CTX-related side effects [44]. However, this finding could also be irrespective of PN and reflect worsening in metabolic functioning of nutrients due to patients’ disease.

Furthermore, an increase in PI correlated significantly with a decrease in limitations of food choice and food quantity (domain digestive symptoms) due to the disease or treatment. There could be a correlation that with fewer digestive problems, there is a higher tolerance for both the amount of food and the choice of food. A further correlation between an increase in PI and an increase in satisfaction with health care showed that patients felt better cared for when they were well nourished. This could be connected to PN, which would be linked with more frequent contact to health care workers. In contrast, there was a significant correlation between an increase in EI and a worsening in flatulence. Flatulence is caused, among other things, by exocrine pancreatic insufficiency, in which the pancreas can no longer produce enough digestive enzymes to split macronutrients [45].

Our analysis is limited by a small sample size, potentially obscuring effects in other domains of QoL. However, this is the first study on this topic in PaCa patients. These patients are difficult to study due to their poor prognosis. Therefore, this study could help in the decision-making process for PN. Allowing nutritional supplements for all patients could impact group differences. Despite this, data completeness was a positive aspect, with only a few isolated missing items, resulting in a reliable analysis.

## Conclusion

Comparing PN to BSNC in advanced PaCa patients undergoing CTx, no significant group differences were observed in QLQ-C30 and QLQ-PAN26 domains, except for worsening social functioning in IG (QLQ-C30). Within-group analysis showed that the item WL improved in the IG (QLQ-PAN26). Correlations, irrespective of groups, indicated mixed effects of increasing PI and EI. However, due to the small sample size, statistical findings should be interpreted with caution. Altogether, deciding on the most suitable nutritional intervention should be individualized, considering patients’ values on social functioning and weight concerns in collaboration with them.

### Supplementary Information

Below is the link to the electronic supplementary material.

Supplementary file1 (DOCX 277 KB)

## Data Availability

Data will be shared upon request.
