# Supplementary material for: Effects of parenteral nutrition + best supportive nutritional care vs. best supportive nutritional care alone on quality of life in patients with pancreatic cancer—a secondary analysis of PANUSCO
Source: Support Care Cancer. 2024 Jun 27;32(7):466. doi: 10.1007/s00520-024-08666-1 (PMC11211116; doi:10.1007/s00520-024-08666-1)

# Additional information

# Supplementary Information

**Fig S I** Study design


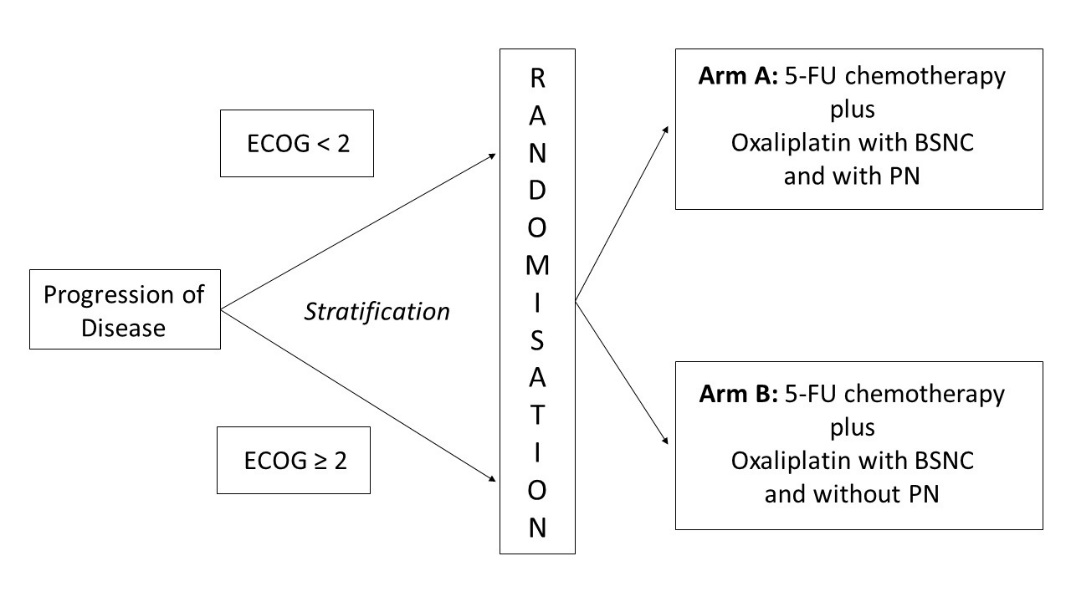


**Tab S I** stopping criteria of both arms were met if two of the following three criteria were present:

| Weight loss | > 2 % within the last seven days or caloric intake ≤ 500 kcal expected within the next five days |
| --- | --- |
| Bio impedance analysis (BIA) phase angle and body cell mass (BCM) | with a deterioration > 10 % (in both parameters) compared to baseline assessment |
| PINI-index | > 10 (only in patients with no sign of acute inflammation). |

**Fig S II** QLQ C30 correlation between change in protein intake (g/kg BW) and change in social functioning

**
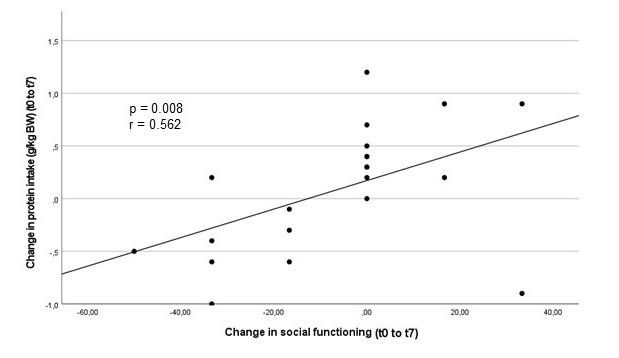
**

**Fig S III** QLQ C30 correlation between change in energy intake and change in role functioning


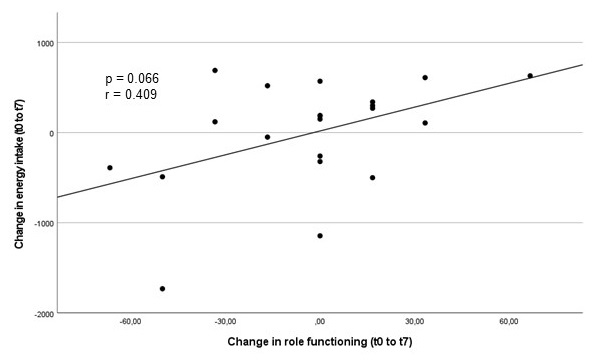


**Fig S IV** QLQ PAN26 correlations between change in protein intake (g/kg BW) and 1) change in planning of activities 2) change in digestive symptoms 3) change in satisfaction with health care 4) change in troubled with side-effects


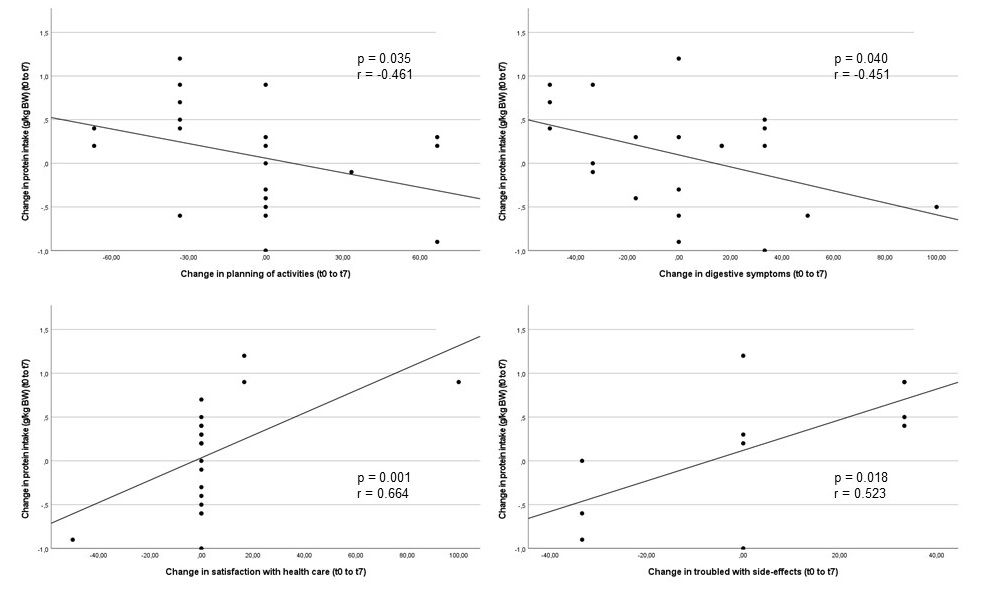


**Fig S V** QLQ PAN26 correlations between change in energy intake and 1) change in planning of activities 2) change in flatulence


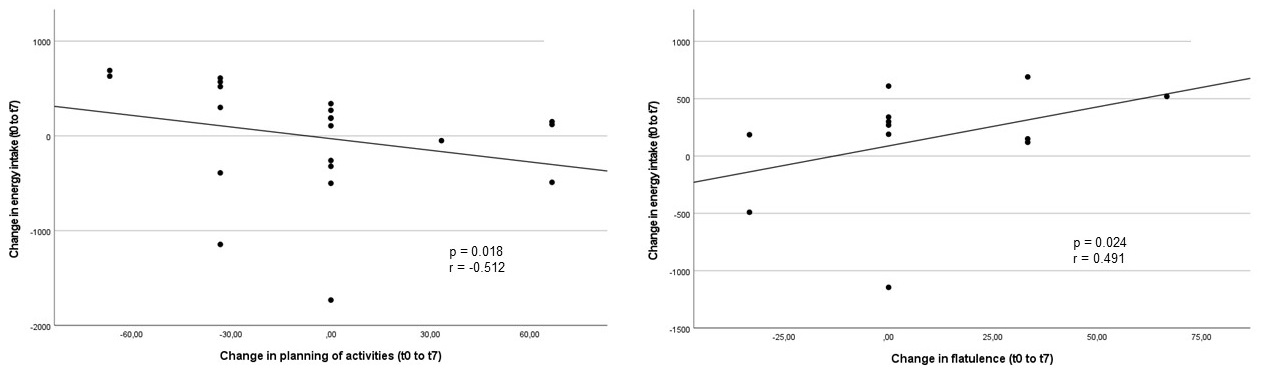

Supplement: Supplementary file 1 — Supplementary file1 (DOCX 277 KB) [file 520_2024_8666_MOESM1_ESM.docx]
